# Finding solid ground: law enforcement, key populations and their health and rights in South Africa

**DOI:** 10.7448/IAS.19.4.20872

**Published:** 2016-07-18

**Authors:** Andrew Scheibe, Simon Howell, Alexandra Müller, Munyaradzi Katumba, Bram Langen, Lillian Artz, Monique Marks

**Affiliations:** 1 Independent Consultant, Cape Town, South Africa; 2 Desmond Tutu HIV Centre, Institute of Infectious Diseases and Molecular Medicine and Department of Medicine, University of Cape Town, Cape Town, South Africa; 3 Centre of Criminology, University of Cape Town, Cape Town, South Africa; 4 Gender, Health and Justice Research Unit, Division of Forensic Medicine, University of Cape Town, Cape Town, South Africa; 5 COC Netherlands, The Netherlands; 6 Urban Futures Centre, Durban University of Technology, Durban, South Africa

**Keywords:** law enforcement, HIV, key populations, relationships, policing, South Africa

## Abstract

**Introduction:**

Sex workers, people who use drugs, men who have sex with men, women who have sex with women and transgender people in South Africa frequently experience high levels of stigma, abuse and discrimination. Evidence suggests that such abuse is sometimes committed by police officers, meaning that those charged with protection are perpetrators. This reinforces cycles of violence, increases the risk of HIV infection, undermines HIV prevention and treatment interventions and violates the constitutional prescriptions that the police are mandated to protect. This paper explores how relationship building can create positive outcomes while taking into account the challenges associated with reforming police strategies in relation to key populations, and vice versa.

**Discussion:**

We argue that relationships between law enforcement agencies and key populations need to be re-examined and reconstituted to enable appropriate responses and services. The antagonistic positioning, “othering” and blame assignment frequently seen in interactions between law enforcement officials and key populations can negatively influence both, albeit for different reasons. In addressing these concerns, we argue that mediation based on consensual dialogue is required, and can be harnessed through a process that highlights points of familiarity that are often shared, but not understood, by both parties. Rather than laying blame, we argue that substantive changes need to be owned and executed by all role-players, informed by a common language that is cognisant of differing perspectives.

**Conclusions:**

Relational approaches can be used to identify programmes that align goals that are part of law enforcement, human rights and public health despite not always being seen as such. Law enforcement champions and representatives of key populations need to be identified and supported to promote interventions that are mutually reinforcing, and address perceived differences by highlighting commonality. Creating opportunities to share experiences in mediation can be beneficial to all role-players. While training is important, it is not a primary mechanism to change behaviour and attitudes.

## Introduction

The nature of relationships and hierarchies between institutions and social groupings in society affects health and wellbeing in complex ways. For those who are already marginalized, such social dynamics can serve to undermine or strengthen their resilience. The effects of such dynamics may be redesigned to mitigate negativity [1]. Here we speak specifically to law enforcement agencies and key populations (KPs), defined by the United Nations Joint Programme on HIV and AIDS (UNAIDS) as social groupings that are among the most likely to be exposed to HIV and who are negatively affected by punitive laws and stigmatizing policies [2,3]. Although the South African Constitution procedurally mandates the freedoms and services needed to support such KPs, both sex work and drug use remain illegal [4–6]. Sex workers (SWs); people who use drugs (PWUDs); gay, bisexual and other men who have sex with men (MSM); lesbian, bisexual and other women who have sex with women (WSW), and transgender people (TG) are at higher risk of HIV infection than the general population [7–12]. However, emerging evidence reveals that they are particularly affected by discursive and physical abuse, stigma, discrimination and exclusion [7,11,13–17]. Moreover, public opinion in general tends to reinforce heteronormative, politically driven and morally based frameworks that have negative health and socio-economic consequences for these groups of people [18–20].

### Engagement between KPs and law enforcement

KPs in South Africa have reported multiple forms of abuse perpetrated by police officers. SWs’ experiences of harassment, assault, rape, extortion and condom confiscation by police officers and the denial of access to medication while in custody are well-documented [21–25]. Similarly, research confirms that stigma and discrimination on the basis of sexual orientation and gender identity by police officers occurs both within communities and in police facilities [16,26–28]. At a national consultation in Cape Town in 2014, PWUDs identified negative engagement with law enforcement as their primary concern [29]. This was confirmed during a 2015 programmatic mapping study [30], and in a report highlighting PWUDs’ experiences including harassment, violence, the confiscation and breakage of injecting equipment, and extortion by law enforcement officers in three cities where a needle and syringe programme is operating [31].

Law enforcement efforts are nonetheless a vital service; thus, working with law enforcement agencies to improve public health in South Africa is crucial [32,33]. Despite limited data on the comparative effectiveness of different law enforcement interventions to reduce HIV-related risks and improve the health outcomes of KPs [32], there are examples of law enforcement champions, training and meaningful engagement that have brought about change [34,35]. Notably, these include the “Pink in Blue Amsterdam Police lesbian, gay, bisexual and transgender (LGBT) Network” (“Roze in Blauw”), which has improved the safety of LGBT people for over 15 years by providing contact points to access police services [36]. Closer to home, in Kenya, the institutionalization of training around sex work, health and rights at the Nyanza Provincial Police Training Centre, which had trained over 600 officers by 2015, has improved relationships, reduced violence and increased access to law enforcement services [35]. In Dar es Salaam, support from the National Police Commissioner and the training of law enforcement officers on harm reduction has led to a recognized reduction in drug-related harm and crime [37].

### Law enforcement reform

Changes in policing practice in South Africa have been slow, despite increasing global emphasis on encouraging environments to eliminate stigma and discrimination and enable access to health services [11,38–40]. Many recorded instances of police practices have humiliated and degraded individuals and purposefully compromised their access to health services, especially KPs [20,25,41,42]. However, these concerns have not received significant national attention; nor have the drivers or determinants, such as the relationships between officers and KPs, come under sufficient scrutiny [42]. Indeed, many past interventions aimed at implementing alternative approaches to deal with KPs have been rejected and ignored, or have not facilitated the redirection of the day-to-day acts and relationships that define policing at the community level. In 2012, for example, significant pressure from civil society was required to obtain authorization from the Deputy Minister of Police to enable 80 police officers to receive sensitivity training around sex work. However, the training has not been scaled up or included in police training curricula [43]. As a result, the antagonistic relationships that heighten the risk of violence and abuse against KPs continue and/or increase [2,24], despite public litigation efforts to reinforce the constitutional rights of citizens and the creation of enabling environments, particularly for SWs and TG people [44–46].

In reviewing the records of problematic events, it is clear that police officers are often the primary responders and representatives of governance, and therefore cannot simply be ignored. Indeed, we argue that not only should law enforcement be included in such measures, but that they could become enablers of more appropriate responses and services, ironically because they frequently engage with and have a unique “understanding” of KPs. This dynamic is clearly reflected in a Durban-based project, discussed below. This understanding could become the basis for supportive interactions that could contribute to an effective HIV response. That said, police officers and agencies do not operate in a vacuum; their structures and organizational cultures may incorporate more widely shared understandings of KPs, gender, violence and other social factors [47,48].

Though a detailed discussion of South African policing “culture” is beyond the scope of this paper, it is critical to note that neither the institutions nor the relationships to which it speaks are stagnant. They are, therefore, sites of potential change [49]. To address these complexities, this paper draws on our experience and knowledge. Authors include South African researchers in the fields of criminology, gender and health, and KP HIV programme implementation, as well as a Dutch organization that has worked with sexual minority groups for over 60 years. We set out to review the literature and reflect on our programmatic and research experiences. We used these activities to explore alternative ways of understanding, communication and collaboration between police and KPs to improve KPs’ health and rights and the operational effectiveness of law enforcement. The paper aims to contribute to an emerging scholarship on the relationships between law enforcement agencies, violence and public health [32].

## Discussion

Effective social and institutional interventions require a combination of processes that include reflective and experiential education programmes, and the mutual commitment of stakeholders [50,51]. Moreover, sustainable change requires that interventions “fit” with relevant constitutional and legal principles [52]. In line with this, the development of supportive relationships between law enforcement agencies and KPs cannot be forced or entered into solely from one perspective or another. Rather, it is to *each other* that such concerns need to be directed and promoted, through the crafting of a common language (and understanding) that is meaningful to those affected. This could engender shared responsibilities, which prevent the cyclical forms of antagonism and violence that undermine or prevent interventions. Moreover, hostile, unsupportive and/or distrusting relationships retard development with deleterious consequences for the health of KPs as well as their behaviours in seeking law enforcement services [38]. It is thus in the interests of both “parties” to find a common language. Such commonality can only be created in spaces in which conversations are bidirectional. Without such shared conversations, power disparate relationships are likely to continue and be reinforced [53]. Such spaces of engagement should therefore encourage honest and non-offensive communication that recognizes uniqueness, divergence in opinions and the capacity for change [54].

The prioritization of similarity rather than difference is a useful focal point [55]. This is evident in the shifts that have already taken place in South African policing agencies in the past two decades around rights, HIV and diversity. For example, the South African Police Service's Code of Conduct reflects the South African Constitution, with members undertaking to “uphold and protect the fundamental rights of every person” [56]. A comprehensive Employee HIV Programme is in place [57], and the South African Police Service has become more responsive to the need for a sensitive approach to HIV [57].

Lessons learnt from the organizational shifts that have occurred could inform police reform in other areas, including KPs. The examples above, acknowledgement of the high levels of alcohol and drug use among police officers [58] and the stated need to prioritize employee wellness, including HIV prevention and treatment [59], provide an opportunity to speak about other concerns. Moreover, in replicating the pre-existing structures that have shown success–and that draw on relational understandings–such interventions can be positioned as efficient, familiar and effective, which may further increase the likelihood of adoption and uptake.

Such engagement and related training is evidently needed, as power differentials between individuals and groups can affect the outcome of engagements, usually in favour of the more powerful party [20]. Despite similar backgrounds, engagements between law enforcement and KPs have, for the most part, been antagonistic with the police often asserting their legal and situational power [43]. It is therefore not surprising that, in June 2015, SWs participating in a workshop in Cape Town, attended by representatives of 26 organizations, reported more instances of harassment, bribery and violence at the hands of law enforcement agents than positive, supportive and respectful engagements [60], a clear reflection of the evidence cited earlier. Equally, KPs’ behaviours towards law enforcement are often hostile and may escalate police aggression [60]. This fuels antagonism and distance between KPs and law enforcement officers, which, in the context of unequal power, can lead to police violence, discrimination and abuse [61].

What can we learn from this? In the first instance, interventions aimed at more sensitive relational outcomes would allow the parties to acknowledge their personal, social and structural challenges [62]. Reflective and candid engagement has a better chance of enhancing “understanding” than laying complaints and demands squarely at the feet of police officers or blaming KPs for a range of social “ills”. The starting premise of the police as the root cause of deteriorating community relations, who trigger or exacerbate the vulnerability of hard-to-reach groups, alienates the police from interventions, disempowers KPs and blocks constructive relational possibilities [63].

Neither the police nor KPs are immune to change, but equally, neither wish to be the target of blame. There is no single police understanding; police officers’ responses to KPs differ and may be incongruous, as is generally the case in the policing of marginalized groups [64]. The practices of the police are shaped by their everyday interactions on the streets and through reflective engagements with KPs, rather than through formal training [65].

### Establishing relationships and creating a change agenda

Below we outline several on-going initiatives that are being led by universities and civil society organizations in South Africa. We believe that these projects offer constructive opportunities to shift relational paradigms between police and KPs.

Since 2014, researchers based at the Urban Futures Centre at the Durban University of Technology have forged close links with police agency units engaged in the policing of drug use and sex work in the city [66]. Over the past year, ethnographic journeys in police vans, discursive workshops and the secondment of law enforcement officers to university spaces have taken place. Civil society organizations experienced in providing HIV-related health services to SWs and PWUDs have co-facilitated workshops where police “thinking,” questions and dilemmas have framed the flow of conversation [43]. Immediacy has been used as a tool to enable open conversations about personal dilemmas, including personal experiences of substance use and living on the street. These efforts have improved trust and led to mutual respect for one another's expert knowledge and have identified alternatives to the “traditional” policing of KPs. Police support for needle and syringe programmes and training opportunities have been discussed. During these discussions police officers highlighted the need for changes in performance management (especially the use of arrest “quotas”) [43,60]. They also highlighted the need for appropriate authorization to prevent “dereliction of duty” [43,60]. A police officer shared the effects of these engagements with an academic researcher: “Since I met you, you have made me softer. I have let go more than 50 drug users that I would otherwise have arrested.” We expect that our efforts to shift policing practice will in turn lead to KPs relating to the police in new ways.

In general, working from the “top-down” with police agencies is critical for organizational change [52]. Law enforcement agencies function and are governed through substantive law, accompanying regulations, National Instructions, Standing Orders and other related policies. Application of and compliance with these “orders” is monitored by police oversight mechanisms and Parliamentary Portfolio Committees, which are hierarchically structured [47]. Although the processes are complex and often slow, paradoxically perhaps, the same top-down, rule-focused nature of these organizations makes police organizational change fairly simple [42]. Memoranda of understanding between law enforcement and civil society organizations and “high-level” police commitment are critical for institutional change and could break the back of “tried and tested traditions,” as has been experienced by researchers in both Cape Town and Durban.

Speaking to this, a pilot training module for law enforcement agencies is planned aimed at improving relationships and engagement with KPs. Attempts to obtain high-level approval from the South African Police Service for this project is still pending, eight months after the submission of the proposal. Efforts are now being channelled through the African Policing Civilian Oversight Forum to access high-level groups of police officers who are committed to police accountability and human rights to catalyze this initiative. Rather than approaching the police directly with a set of tools for additional sensitization training, we argue that it is more strategic to create change that aligns with police concerns and commitments to policing accountability that is itself oriented towards the affirmation of human rights [52]. By engaging the police from a police oversight perspective, we hope to promote understanding and realign relations between law enforcement and KPs with broader strategies aimed at more accountable, effective and just policing practices. The training aims to address an identified need [67] even though the effect of formal training is limited and training lessons often shift dramatically when police are in their work environment [68]. As such, training will complement on-going interventions to improve the health and rights of KPs in South Africa. These include the documentation of violations experienced by KPs and efforts to increase access to justice [31,69,70]; peer-based KPs’ rights literacy activities; capacity building of KP organizations and strategic litigation [30,39,60,71], measures that are being implemented by other civil society organizations. These strategies are recommended by UNAIDS to address stigma, discrimination and increase access to justice in national HIV responses [72].

KPs and law enforcement officers confront demands from their peers and organizational “homes.” In the case of police officials, performance indices may impose “arrest quotas” that drive behaviours that target KPs [40]. Any change in the processes within law enforcement agencies must, therefore, be foregrounded and framed by parallel efforts to address these structural mandates and concerns, in order to open the space in which mutually advantageous relationships can exist. Similarly, some KPs may be antagonistic to any form of positive engagement with the police [42]. This, too, needs to be acknowledged and addressed with reason. Although the decriminalization of drug use and sex work, as recommended by the World Health Organization [73], is ultimately required to maximize the health, rights and wellbeing of KPs in South Africa, interim measures and the development of more effective intervention strategies remain important.

## Conclusions

UNAIDS recommends training, increased access to legal services, improved rights literacy and policy change to address stigma, discrimination and barriers to justice in national HIV responses [72]. We argue that structural constraints – particularly legislation, performance management, accountability mechanisms, training and the physical conditions under which police work – need to be communicated to those who have the capacity and power to bring about change within law enforcement structures, while also working with street level law enforcement officers.

The effectiveness of planned interventions that improve the relationships between law enforcement and KPs, and ultimately the health outcomes of KPs, need to be evaluated to inform future police and health policy reform. In the interim, we suggest a few processes towards improving the health and rights of KPs in South Africa.

In developing a framework for implementation, first, negating “othering” is most likely to result from deliberative forums in which the constraints and possibilities of all groupings are brought to the fore and openly discussed. Solutions that benefit all parties should be identified as well as the constraints in implementing these solutions. Bringing law enforcement officers who work on the streets together with KPs to find innovative solutions is a powerful starting point. Human rights, public health and risk reduction for all should be at the centre of such engagements. Universities and non-governmental organizations are well placed to facilitate such engagements.

Second, it is important to identify law enforcement officers who are champions of human rights-oriented policing, public health access and harm reduction. These champions should come from the apex of the organization as well as from the ranks of police officers who work on the street. Support should be mobilized from significant individuals and organizations for these officers to openly discuss their alternative positions and concerns. The champions would ideally work collaboratively with KPs and public health professionals to find shared agendas and workable interventions. This could be further bolstered by creating a shared language that allows for effective communication; safety outcomes, which include reducing public health risks, are terminologies that are fundamental to police and KPs.

Third, contrary to conventional thinking, it is important to recognize that changes in policy and training, although critical to long-term and sustainable police organizational change will not on their own lead to the required or desired shifts in daily behaviour. Rather, training is a tool in the structural field of policing that is necessary to enable and support daily behavioural change [74]. However, the real impetus for change in the habitus (or everyday responses and behaviour) is far more dependent on the basic assumptions that police hold about particular social groupings, forged while on the street doing police work and through facilitated deliberations and engagements that often take place outside of formal training programmes.

Finally, law enforcement agencies and individuals should be exposed to those who have been at the forefront of promoting human rights, public health and harm reduction based approaches. Exposure to peer organizations – through discussions, international site visits, and digital and social media – would go a long way in assisting them to find legitimacy and resonance.
